# Limb Salvage Surgery With Mega-Prosthesis in a Case of Chondrosarcoma: A Case Report

**DOI:** 10.7759/cureus.28449

**Published:** 2022-08-26

**Authors:** Ankur Salwan, Gajanan L Pisulkar, Shounak Taywade, Vivek H Jadawala, Amit Saoji

**Affiliations:** 1 Department of Orthopaedic Surgery, Datta Meghe Institute of Medical Sciences, Wardha, IND

**Keywords:** clear cell, amputation, proximal tibia, tumor, chondrosarcoma

## Abstract

Chondrosarcoma is a kind of bone tumor that can be anywhere in the body but most commonly affects the pelvis, glenohumeral joint, proximal femur, and proximal one-third of the tibia including condyles. It accounts for 20-25% of all bone sarcomas. Chondrosarcomas with clear cell variants are extremely uncommon, making up just around 6% of all cases. We are presenting a case of a 52-year-old male with a bony lesion over the epiphysis of the left tibia. He was managed with resection of the tumor followed by a limb salvage procedure with mega-prosthesis. Chondrosarcoma affects men in the third to fourth decades of their life more commonly than females. Long-standing localized pain over a prolonged duration is the most common presenting symptom. There are various treatment modalities available for clear cell chondrosarcoma, ranging from wide local resection and intralesional therapy to amputation. The decision of tumor resection followed by prosthesis was chosen over amputation here, as the patient had the lesion for 2 years and there were no signs suggestive of metastasis after thorough screening. Limb salvage gave a better outcome for the patient in our study. A large-segment prosthesis is a suitable reconstructive alternative to amputation. At the majority of the anatomical sites where the prosthesis was employed, the functional results were good or exceptional after this type of treatment. The patient now has a functional limb and is able to resume his life as before, making mega-prosthesis a better alternative and treatment of choice for patients with large lesions.

## Introduction

Chondrosarcoma, a malignant bone tumor that arises from cartilage-forming cells, is the second most prevalent primary bone cancer behind osteosarcoma. The presence of cartilage-forming malignant cells without direct osteoid development distinguishes it from other primary bone cancers. It is divided into four subtypes: conventional, dedifferentiated, mesenchymal, and clear-cell chondrosarcoma. The clear cell subtype is uncommon and makes up around 2% of all chondrosarcomas. It occurs most frequently near the end of long bones, especially in the epiphysis, but the metaphysis and diaphysis may also be affected. Men in their third to fourth decades of life are typically affected [1-2]. The lesion is usually characterized by well-differentiated tumor cells (low-grade) based on histological changes. It is distinguished by the presence of transparent cytoplasm, small fragments of a heavily calcified matrix, and multinucleated osteoclast-like giant cells [3]. The most prevalent location of this tumor is the epiphysis of the long bones such as the proximal humerus and proximal femur. According to the majority of authors, wide resection of the tumor is the best choice of treatment modality in long bones [4]. After five years, the survival rate is expected to be 92% [5-6]. Chemotherapy and radiation therapy are ineffective against low-grade chondrosarcomas, hence surgical treatment is the only option. Because of the tumor's intra-medullary location, the surgical concepts include obtaining a large bone border while conserving anatomic tissues for function.

Following a wide-margin resection of the tumor, reconstruction requires knee arthroplasty and much of the proximal tibia, which may need a mega-prosthesis, an allograft, or an arthrodesis. Arthrodesis is a much better and more recommended alternative for patients than amputation because it gives them a functional limb. Patients choose a mega-prosthesis because it replaces both joint and bone loss with a hinged knee joint that includes a changeable length of implant stem and provides a variety of options for better mobility and patient specificity. These prostheses can be consumer-specific or modular and come in a variety of sizes. It is becoming more popular as a limb-salvage procedure.

## Case presentation

A 52-year-old male, a driver by occupation, came to the outpatient department of our tertiary care hospital with complaints of pain and swelling over the left knee joint for 2 years. At the initial stage, the swelling was of small size, and then there was a gradual increase in the size of the swelling, which was associated with restriction of movement of the left knee joint. There were no aggravating or relieving factors except that the pain increased with activity and decreased with rest; there was no fever, redness, or discharge; and there was no history of significant weight loss. There was no history of locking of the knee joint.

**Figure 1:**
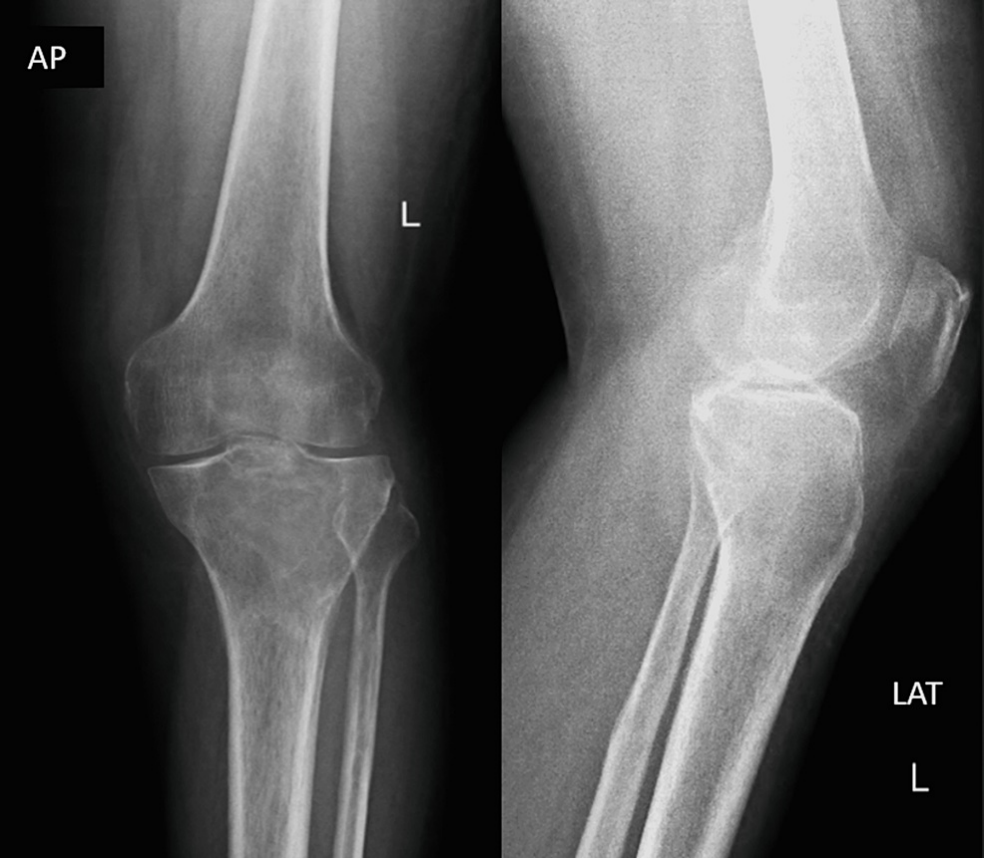
Radiograph suggests a lytic lesion in the proximal aspect of the tibia involving the epiphyseal-metaphyseal junction

**Figure 2:**
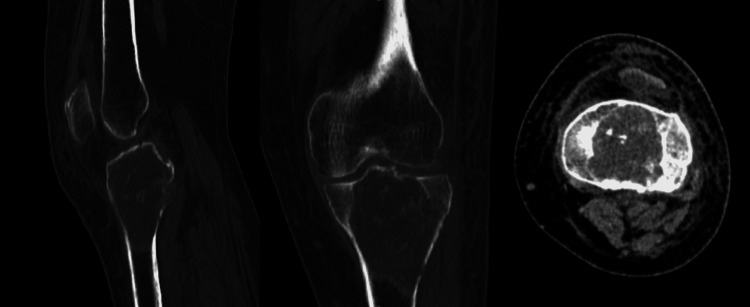
CT scan suggests a lytic lesion in the proximal tibia with areas of cortical erosion

**Figure 3:**
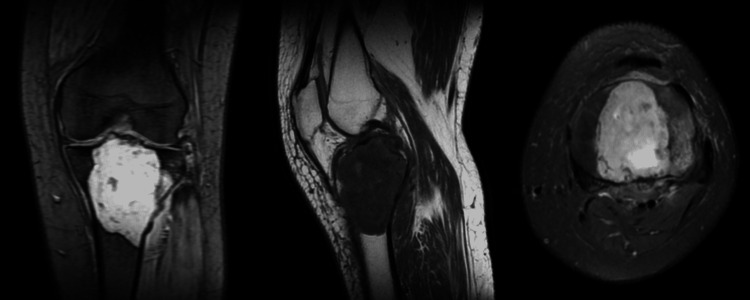
MRI scan suggestive of eccentric soft tissue hyperintense lesion in tibial condyle with peripheral irregular enhancements

On physical examination, the skin over the knee joint was shiny with engorged veins with diffuse swelling over the knee joint. The affected knee had restricted mobility. Tenderness was around the tibial plateau. There was no local rise in temperatures. No ligament laxity was noted. There was no flexion or extension deformity. Distal pulsations were felt. There was no involvement of the common peroneal nerve. The patient had active toe and ankle movements. There was no vascular compromise. The patient used to walk with the help of a stick. Pre-operative radiological investigations were done, in which the x-ray (Figure 1) shows a lytic lesion in the proximal tibia. A further CT scan (Figure 2) was done to look for an intra-articular extension. The CT scan shows a well-defined expansile lytic region with areas of cortical erosion involving the meta-diaphyseal region with subarticular extension. The lesion approximately measures 7.2X5.8X5.1 cm. MRI (Figure 3) was done to rule out soft tissue involvement in the adjacent, which was suggestive of a locally aggressive tumor.

**Figure 4:**
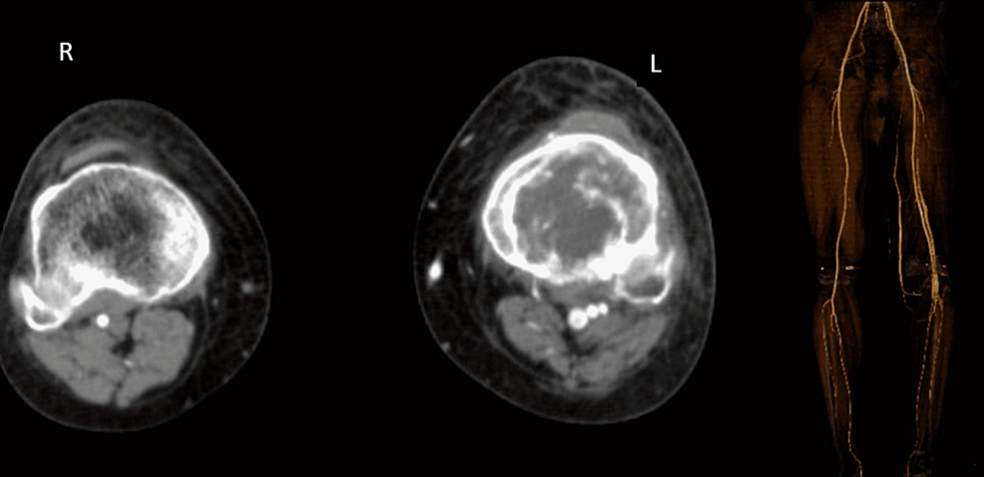
CT angiography suggestive of a tumor extending up to the anterior aspect of the popliteal vessel with no involvement of the vessels

As the decision of excision was planned based on the size of the lesion, A CT angiography was done to rule out vascular involvement and to see the collaterals (Figure 4).

Preoperatively CT-guided biopsy and the histopathology report were suggestive of chondromyxoid fibroma/myxoid change in a giant cell tumor on initial reports, which were again sent for histopathological review given that the presentation of MRI showed the clear cell variant of chondrosarcoma, as this variant of chondrosarcoma mimics giant cell tumor.

Operative procedure

**Figure 5:**
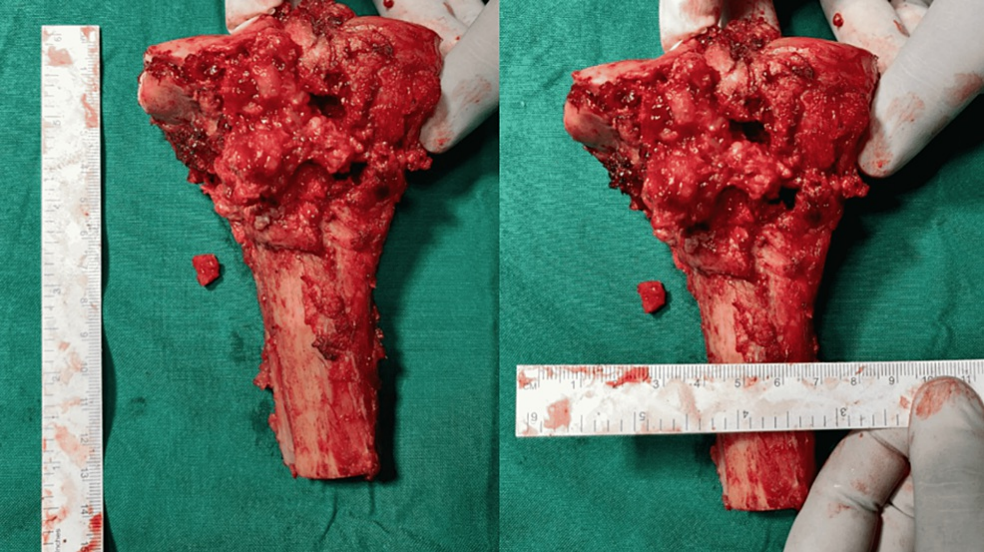
Resected part of the proximal tibia with a healthy tumor-free margin

**Figure 6:**
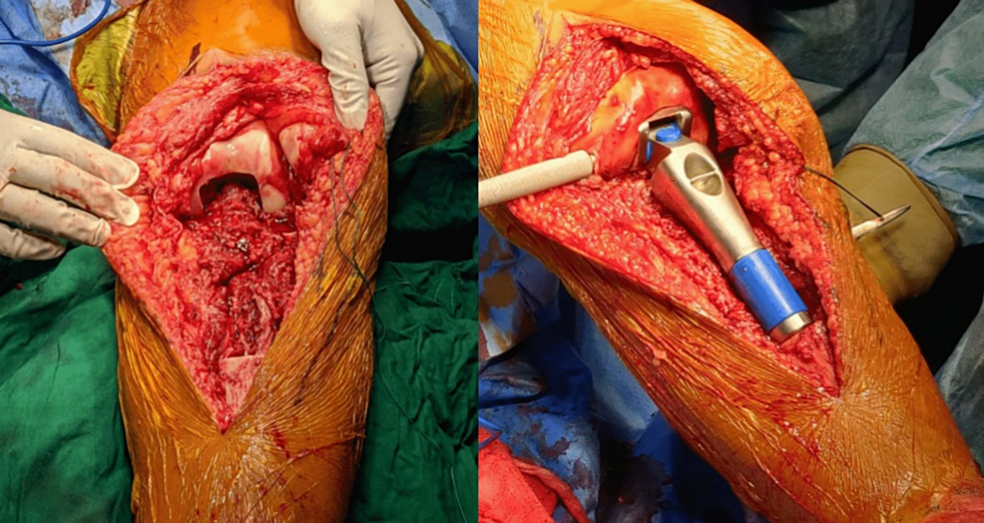
Femoral preparation and placement of prostheses in the femoral and tibial shafts

The patient was planned for proximal tibial resection and reconstruction using a mega-prosthesis. Standard cleaning and draping protocols were followed, and a tourniquet was applied The incision, joint line, and tibia resection level were marked based on CT scan findings. An incision was made along the marking, dissection was carried out to raise the anterior flap, the knee joint was opened, and the patellar tendon was cut with a suitable margin from the tibial disease. The posterior flap was raised by sharp dissection, the pes anserinus was incised from the tibia, and the joint capsule was incised at the meniscofemoral ligament level. Dissection was carried out over the posterior capsule. The knee joint is flexed by everting the patella. The cruciate ligaments were cut, and the lateral collateral ligament and the lateral joint capsule were incised. The dissection was carried out in the posterior plane between the popliteal vessel and the joint capsule, and the remaining posterior capsule was cut carefully. The main neurovascular bundle was exposed and dissected. The feeders to the tumor were identified, ligated, and cut. The level of tibial resection was marked, a perpendicular tibia osteotomy was done, and the resected part with free margins (Figure 5) was separated. Femoral and tibial preparation was done, and the trial components were inserted and stability checked. After trial preparation (Figure 6), the mega-prosthesis was inserted. The patellar tendon was then inserted with the help of an ethibond suture onto the notch provided on the anterior part of the tibial component. The medial gastrocnemius flap was then rotated to cover the implant and was sutured to the collateral ligament, the retinaculum, and the patella tendon. The prosthesis was completely covered by healthy muscle, the subcutaneous tissue was then closed over the drain, and the skin closure was then completed.

 

Postoperative

**Figure 7:**
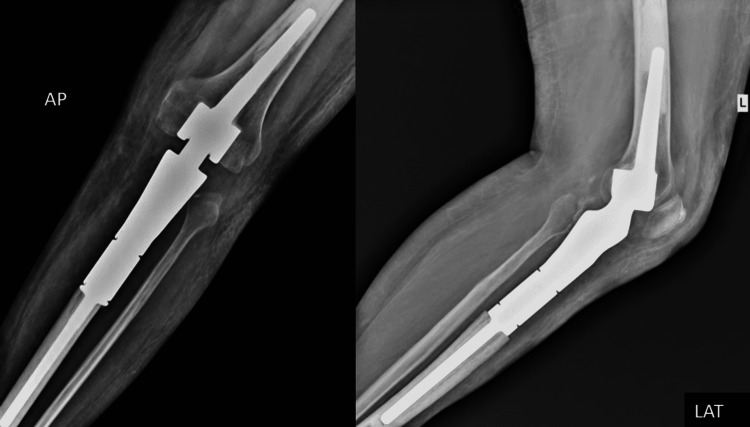
Postoperative x-ray AP and lateral view with mega-prosthesis in-situ AP: anteroposterior

**Figure 8:**
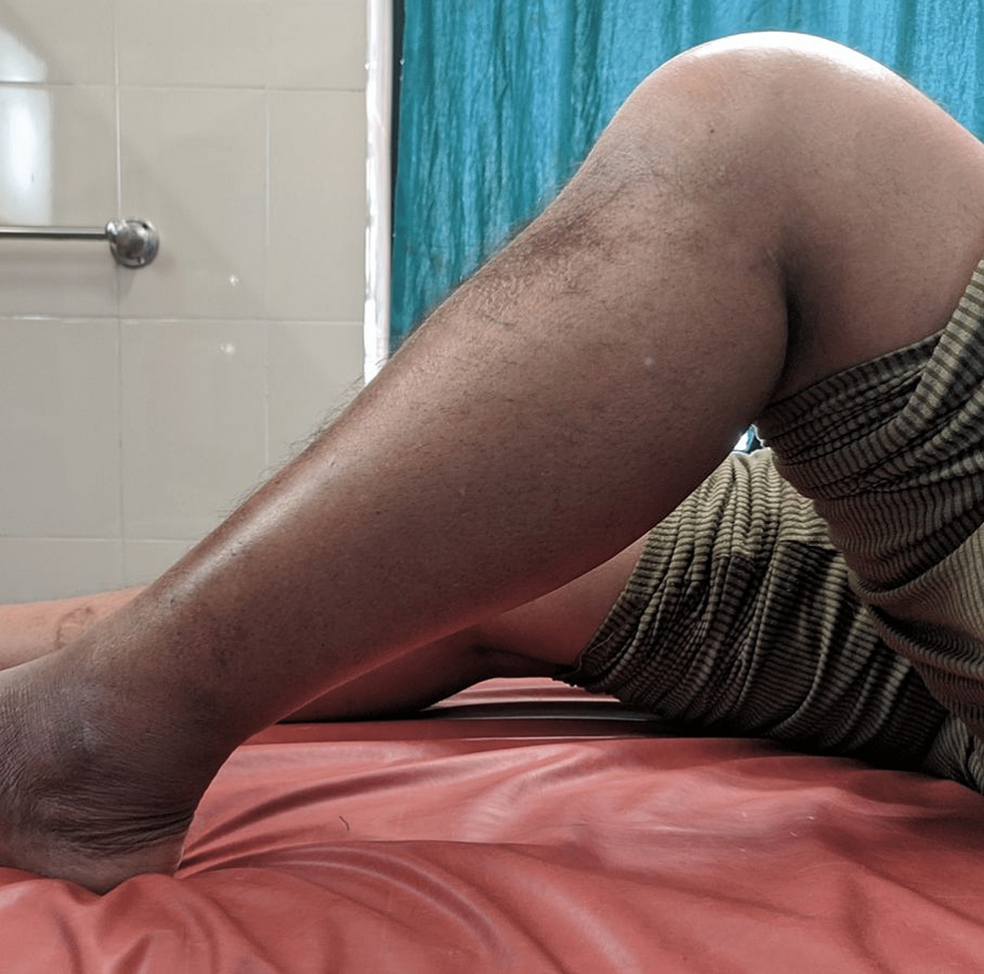
Knee range of movement after two years with a healthy surgical site

The patient was given a long knee brace and the proper antibiotic and analgesic cover. A postop x-ray (Figure 7) was done, which showed satisfactory reduction and mega-prosthesis placement. Physiotherapy started in the form of guarded passive knee range of motion (ROM) exercises on day 4. The non-weight-bearing mobilization with a walker started and the postop histopathology report showed clear cell chondrosarcoma; this was later confirmed on immunohistochemistry. Suture removal was done on postoperative day (POD) 12, full weight-bearing with the help of a walker was started, and active knee ROM was started. After one month of follow-up, the patient was walking with a painless limb with full knee ROM and with the support of a stick. The patient is advised to undergo chemo and radiotherapy with continued screening for metastasis. Now, after 2 years, the patient has resumed his daily routines with full range of movement (Figure 8), and no complications have been reported to date.

## Discussion

Chondrosarcoma (clear cell type) is a low-grade malignant variant of bone tumors. It is most common in the third to fourth decades of life, with male predominance. Long-term, unspecific local pain is a common symptom. Bjorgsson et al. found that 55% of their 47 patients had been experiencing symptoms for more than 1 year, which they attributed to the tumor’s gradual growth [1-3]. According to scientists, there is a subset of CCC that has a poor prognosis that is not predicted by traditional histopathological analysis. Wide tumor resection is the treatment of choice. According to Bjorgsson et al., the tumor recurrence rate with this therapy technique is 15% [3]. Several studies have looked into the debate between limb salvage and amputation. According to Simon et al., while patients with limb salvage had a higher chance of local recurrence, there was no overall difference in survival [7].

Here also, the decision of tumor resection followed by prosthesis was chosen over amputation given the involvement of the whole of the proximal tibia along with its articulating surface, leaving the options of local adjuvant therapy. The patient had had a lesion for 2 years, and there were no signs of metastasis. Shemesh SS et al., in their meta-analysis, concluded that when compared to wide local excision, intralesional therapy produced superior functional outcomes and had few complications. For low-grade chondrosarcomas, there was no significant difference in the probability of local recurrence or metastasis for central chondrosarcomas [8]. Similarly, Dierselhuis EF et al., in their meta-analysis, concluded that outcomes and complications are few in the intralesional group rather than the surgery group, but it is only limited to the low grade, that is, grade 1, but it is hard to grade these [9]. Local relapse, on the other hand, does not invariably lead to metastases followed by death. The prognosis for patients varies depending on the tumor grade. Following a local relapse, limb salvage becomes more difficult, and several problems may arise. Hence, the treatment of choice is wide local excision, as the recurrence rate is high among clear cell chondrosarcoma patients.

Limb salvage surgery is now the preferred treatment option for tumors such as Ewing sarcoma and osteosarcoma rather than amputation. Long-term life expectancy has increased with the invention of chemotherapy. After wide local resection of the tumor at the ends of long bones, the large bone defect is reconstructed either with arthrodesis, allograft, bone grafting, temporary cementing, or the use of special prostheses for tumors. The allograft provides restoration of bone stock and better patellar tendon re-attachment though collapse and infection still remain a concern as described by Clohisy DR et al. [10]. Enneking WF et al., in their study on 20 patients, did resection arthrodesis by using an intramedullary rod and local bone graft for lesions around the knee joint, providing a stable but stiff knee [11]. In a study by Sanjay BK et al., 33 patients with bone tumors were treated by resection of the growing tumor and reconstruction. They concluded that in more than three-quarters of the distal femur reconstruction instances, excellent or good functional results were achieved, although it should be used with caution in the proximal tibia and proximal femur [12]. Initial results from Grimmer et al.'s study of 151 patients with proximal tibial tumor endoprosthesis showed a 33% infection rate and wound dehiscence. This was reduced to 12% when the prosthesis was covered with a gastrocnemius flap [13].

The main concerns after tibial resection are poor tibial muscle and skin cover, patellar tendon reattachment, and damage to the popliteal vessels and common peroneal nerve. The main reason for the better results in distal femur resection was good soft tissue cover, but this problem can be avoided by using a gastrocnemius flap to cover the prosthesis and provide a good floor for the skin cover. Additionally, skin grafting can be done to avoid skin tension.

Another major concern is the patellar tendon, as adherence to metal is a major concern. A tendon can be attached to the gastrocnemius flap. Petschnig et al. studied the results of various surgical procedures for rebuilding the extension apparatus after proximal tibia excision and tumor prosthesis implantation in 17 patients by three different methods, of which suturing with the patellar tendon with gastrocnemius or to the transposed fibula was successful for achieving active extension [14].

Joint replacement after tumor resection from the proximal tibia poses more challenges as compared to tumors of the distal femur, but this can be taken care of by using a gastrocnemius flap, good extensor mechanism reconstruction, and avoiding skin tension. Cipriano CA et al. concluded that the gastrocnemius flap aids in the extension mechanism and wound healing [15]. Because chondrosarcoma is a low-grade malignancy, the patient will need to be screened for metastasis and local recurrences.

## Conclusions

In this study, the patient was given a chance of limb salvage surgery as compared to what was advised initially. Histopathologically, giant cell tumors often mimic chondrosarcomas. The patient now has a functional limb with a good range of motion, and he can bear weight on the operated limb and walk. There are no complications related to surgery after 2 years of follow-up such as wound dehiscence, infection, or extensor lag. The patient has resumed his life as before with a functional limb. With the prosthesis, the functional results and capacities we achieved are outstanding. By taking appropriate measures and proper preoperative evaluation and by raising a good flap for healing, it is possible to keep the complication rate low. The outcomes of limb salvage treatments are better than or comparable to those of initial amputation, as there is no psychological factor involved and patient acceptance is better. However, rather than limb salvage, the treatment's main goal is local tumor management. The patient does not profit from the preservation of a limb with a locally recurring malignancy. However, a large lesion with no vascular involvement and a good functional outcome prompted us to pursue limb salvage using a modular mega prosthesis in our study at a tertiary care hospital and the poor economic background of the patient.
